# Supplementary material for: FastqPuri: high-performance preprocessing of RNA-seq data
Source: BMC Bioinformatics. 2019 May 3;20:226. doi: 10.1186/s12859-019-2799-0 (PMC6500068; doi:10.1186/s12859-019-2799-0)
Supplement: Supplementary file 2 — Archive of FastqPuri. Archive containing all files needed to install and run FastqPuri v1.0.6. Date stamp March 22, 2019. (GZ 47,819 kb) [file 12859_2019_2799_MOESM2_ESM.gz › FastqPuri-1.0.6/html/fq__read_8c.html]

FastqPuri: src/fq\_read.c File Reference


FastqPuri


- src

Functions

fq\_read.c File Reference

fastq entries manipulations (read/write)
More...

`#include <string.h>`  
`#include <stdio.h>`  
`#include <stdlib.h>`  
`#include "fq_read.h"`  
`#include "str_manip.h"`

Include dependency graph for fq\_read.c:

|  |  |
| --- | --- |
| Functions | |
| int | get\_fqread (Fq\_read \*seq, char \*buffer, int pos1, int pos2, int nline, int read\_len, int filter) |
|  | reads fastq line from a buffer More... |
|  | |
| int | string\_seq (Fq\_read \*seq, char \*char\_seq) |
|  | writes the fq entry in a string More... |
|  | |

## Detailed Description

fastq entries manipulations (read/write)

Author
:   Paula Perez paula.nosp@m.pere.nosp@m.zrubi.nosp@m.o@gm.nosp@m.ail.c.nosp@m.om

Date
:   03.08.2017

## Function Documentation

## ◆ get\_fqread()

|  |  |  |  |
| --- | --- | --- | --- |
| int get\_fqread | ( | Fq\_read \* | *seq*, |
|  |  | char \* | *buffer*, |
|  |  | int | *pos1*, |
|  |  | int | *pos2*, |
|  |  | int | *nline*, |
|  |  | int | *read\_len*, |
|  |  | int | *filter* |
|  | ) |  |  |

reads fastq line from a buffer

a fastq line is read from a buffer and the relevant information is stored in a structure **Fq\_read**. Depending on the value of **filter**, information about whether the read was trimmed is stored.

Parameters
:   |  |  |
    | --- | --- |
    | seq | pointer to **Fq\_read**, where the info will be stored. |
    | buffer | variable where the file being read is stored. |
    | pos1 | buffer start position of the line. |
    | pos2 | buffer end position of the line. |
    | nline | file line number being read. |
    | read\_len | predefined read length |
    | filter | 0 original file, 1 file filtered with filter\_trim, 2 file filtered with another tool |

## ◆ string\_seq()

|  |  |  |  |
| --- | --- | --- | --- |
| int string\_seq | ( | Fq\_read \* | *seq*, |
|  |  | char \* | *char\_seq* |
|  | ) |  |  |

writes the fq entry in a string

Parameters
:   |  |  |
    | --- | --- |
    | seq | pointer to **Fq\_read**, where the info will be stored. |
    | char\_seq | pointer to buffer, where the sequence will be stored |

Warning
:   change the call to sprintf to snprintf


---

Generated on Mon Mar 19 2018 23:42:01 for FastqPuri by  

 1.8.14
